# Diversity of Nematode Microbial Antagonists from Algeria Shows Occurrence of Nematotoxic *Trichoderma* spp.

**DOI:** 10.3390/plants9080941

**Published:** 2020-07-24

**Authors:** Nawal Benttoumi, Mariantonietta Colagiero, Samira Sellami, Houda Boureghda, Abdelaziz Keddad, Aurelio Ciancio

**Affiliations:** 1 Laboratory of Phytopathology and Molecular Biology, Department of Botany, Higher National School of Agronomy (ENSA), El-Harrach 16004, Algeria; n.bentoumi@edu.ensa.dz (N.B.); s.sellami@hotmail.fr (S.S.); hou.boureghda@gmail.com (H.B.); a.kedad@ensa.dz (A.K.); 2 Consiglio Nazionale delle Ricerche, Istituto per la Protezione Sostenibile delle Piante, Via G. Amendola 122/D, 70126 Bari, Italy; aurelio.ciancio@ipsp.cnr.it

**Keywords:** biocontrol, *Globodera rostochiensis*, *Meloidogyne*, *Trichoderma*, *Fusarium*, TRI gene, trichothecenes

## Abstract

Fungi and bacteria associated to phytoparasitic nematodes *Globodera rostochiensis* and *Meloidogyne* spp. in Algeria were identified and characterized. *Trichoderma* spp. showed the highest prevalence in the cysts of *G. rostochiensis*. A number of isolates were identified through PCR amplification and the sequencing of the internal transcribed spacer (ITS)1-2 and *Rpb2* gene regions. The most represented species were *T. harzianum* and *T. afroharzianum*. The latter and *T. hirsutum* were reported for the first time in Algeria. *Fusarium* spp., including *F. oxysporum* and *F. solani*, comprised a second group of fungi found in cysts. Taxa associated to females of *Meloidogyne* spp. included *T. harzianum*, *Fusarium* spp. and other hyphomycetes. To assess the efficacy of *Trichoderma* spp., two assays were carried out in vitro with the culture filtrates of two *T. afroharzianum* and *T*. *harzianum* isolates, to check their toxicity versus the second stage juveniles of *M. incognita*. After 24–48 h exposure, a mortality significantly higher than the control was observed for both filtrates at 1% dilutions. The *TRI* genes involved in the production of trichothecenes were also amplified with the PCR from some *Trichoderma* spp. isolates and sequenced, supporting a putative role in nematode toxicity. Bacteria isolated from the cysts of *G. rostochiensis* included *Brucella,* *Rhizobium, Stenotrophomonas* and *Bacillus* spp., identified through 16S rRNA gene sequencing. The potential of the microbial isolates identified and their mechanisms of action are discussed, as part of a sustainable nematode management strategy.

## 1. Introduction

Major plant pests such as plant-parasitic nematodes (PPNs) induce severe annual losses in agricultural and industrial crops, affecting their productivity worldwide. Species of the genera *Meloidogyne* (root-knot nematodes), *Heterodera* or *Globodera* (cyst nematodes), and *Pratylenchus* (lesion nematodes) are among the most economically important PPNs, due to the damage, host ranges and persistence in soil. Conventional pest management and the related agricultural practices based on pesticides are affected by the insurgence of resistance in pests or pathogens and by a number of environmental risks, including soil and water contamination. Moreover, many registered products applied for PPN control in conventional agriculture have been withdrawn from the market, because of their low specificity, toxicity and environmental impact [1].

The management of PPNs is challenging, in particular in sustainable and organic agriculture. In recent years, there have been increasing efforts to develop sustainable management approaches based on biocontrol agents, including the use of their metabolites, in search of efficient alternatives to conventional control practices. However, PPNs have evolved a long-term resilience towards plant immune reactions [2], as well as versus other ecosystem regulating factors, including microbial antagonists. Several studies addressed the direct or indirect antagonistic activity exerted towards PPNs by soil fungi, including the effect of metabolites and of rhizosphere interactions [3,4,5,6].

A number of fungal species have been used and applied in recent decades for PPN biocontrol, including hyphomycetes such as *Pochonia chlamydosporia*, *Arthrobotrys* or *Trichoderma* spp. [3,7]. A selection of isolates was considered indispensable, in many cases, to achieve an acceptable biocontrol level, often due to biochemical specialization or virulence [3].

Adding to fungi, several genera of soil bacteria such as members of genera *Pasteuria*, *Bacillus* and *Pseudomonas* act as natural PPN regulators, with different levels of specificity and efficacy [8,9].

In Algeria, *Globodera* and *Meloidogyne* spp. are the most dangerous PPNs, causing a severe impact on several crops [10,11]. Their management is mainly based on nematicides including fumigants and organophosphates. Management options are increasingly oriented towards methods which respect the environment, food safety, human and animal health. Nematode parasitic bacteria and antagonistic fungi are the microorganisms most studied in PPN biological control. In recent years, however, emphasis has been placed also on the use of organic amendments, plant extracts or their derived products [12,13]. The effectiveness of several antagonistic fungi has been reported against *Meloidogyne* and *Globodera* [14,15,16,17,18,19]. Apart from *P. chlamydosporia*, the candidates for the development of commercial bioformulations include species of *Trichoderma* characterized by a range of antagonistic effects, also related to the production of active metabolites. *Trichoderma harzianum* proved to be an effective biocontrol agent of root-knot nematodes, inducing immobilization through enhanced proteolytic activities [20]. Formulates of *T. asperellum* and *T. harzianum* induced resistance to *Meloidogyne incognita* in tomato plants and reduced the nematode eggs and egg masses, with an effect that was additive to that of the tomato resistance gene Mi-1.2 [21].

This study aimed at identifying the isolates of PPN antagonists, with an agronomic interest and a biocontrol potential, naturally present in nematode-infested soil and root microcosms in Algeria. In particular, we investigated the occurrence of microbial antagonists associated to *G. rostochiensis* or *Meloidogyne* spp. in farms among different areas of Algeria, and the occurrence of nematotoxic metabolites in some of them. First, the data on the species diversity and the nematocidal potential of some isolates are herein provided.

## 2. Results

Most fungi isolated from or associated to PPNs in Algeria included members of the genera *Trichoderma* and *Fusarium*, followed by a few other hyphomycete taxa (Table 1). *Trichoderma* spp. (27%) were found in the cysts of *G. rostochiensis* as well as in soil or females of *Meloidogyne* sp. These genera showed a higher frequency in cysts (60% of isolates). Among *Fusarium* spp. (24%), most isolates proceeded from the cysts of *G. rostochiensis*, with *F. oxysporum* (7%) as the most common species (Table 1).

**Figure 1:**
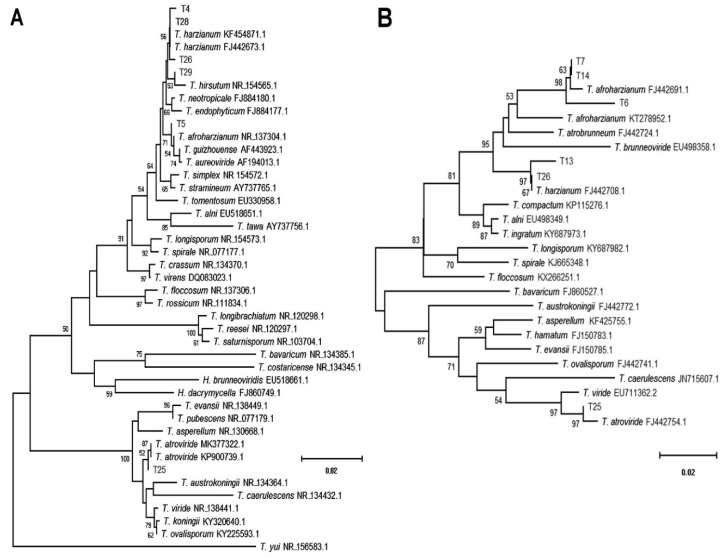
The phylogenetic distance trees of *Trichoderma* spp. isolates with the closest species (including *Hypocrea* spp. anamorphs), based on sequenced PCR products and available GenBank accessions. Dendrograms inferred with MEGA X, applying the neighbor-joining and Jukes–Cantor methods to the aligned sequences of the ITS1-2 (**A**) and *Rpb2* (**B**) gene regions. The percent of trees in which the associated taxa clustered together in the bootstrap test (100 replicates) are shown next to the branches (threshold: 0.5). Optimal trees, with the sum of the branch lengths = 0.54914559 (**A**) and 0.56187331 (**B**), are drawn to scale, in the number of base substitutions per site.

The PCR products of the internal transcribed spacer (ITS) and *Rpb2* gene sequences were obtained for 26 out of 28 *Trichoderma* isolates. Their sequencing allowed the construction of two corresponding and consistent dendrograms, based on the neighbor-joining method (Figure 1A,B).

The clusterings of the sequences produced with those of the known species allowed for the identification of all isolates but T14 (with conflicting assignments, see Table 1) and T15 (not amplified). *Trichoderma harzianum* and *T*. *afroharzianum* were the most common species encountered (representing 69% and 23% of isolates, respectively), as shown by the 100% identity of isolates (not included in the dendrograms). These two species were followed by *T. hirsutum* and *T. atroviride*, which appeared with a much lower frequency, corresponding to one isolate each (Table 1, Figure 1A,B).

**Figure 2:**
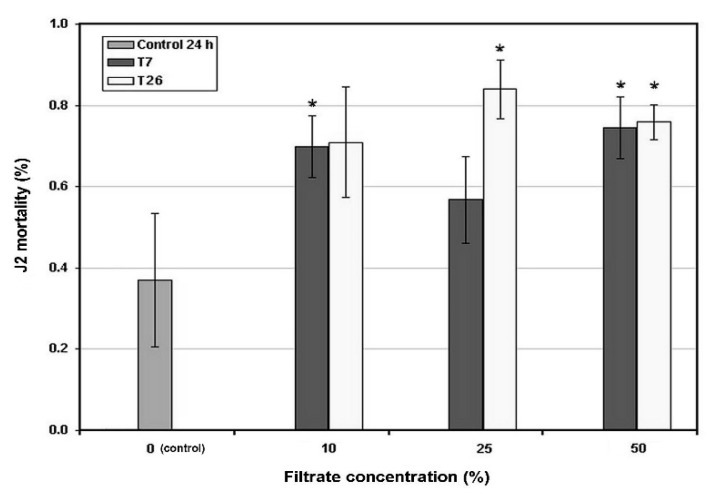
The in vitro effect of the culture filtrates of *Trichoderma afroharzianum* isolate T7 and *T. harzianum* isolate T26 on *Meloidogyne incognita* second stage juveniles (J2), after 24 h exposure at 26 °C, at different concentrations. The control was a 50% dilution of the culture medium used for fungal growth, without the filtrate addition. Bars show the means ± SD. Asterisks show the means significantly different from the control (Student’s *t* test, *p* ≤ 0.05).

**Figure 3:**
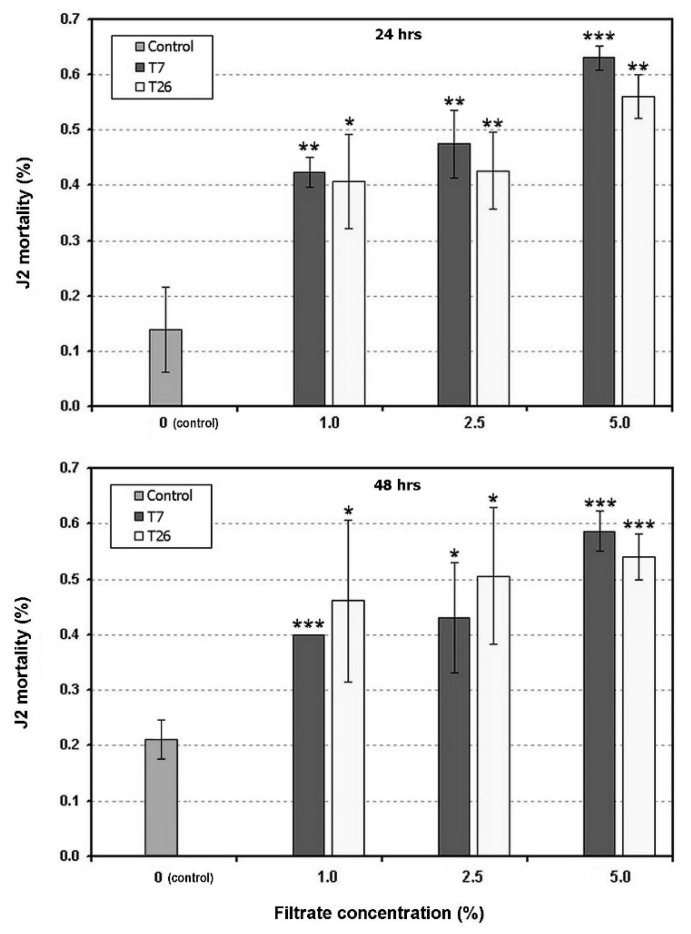
In vitro toxicity of the culture filtrates of the *Trichoderma afroharzianum* isolate T7 and *T. harzianumi* solate T26 on *Meloidogyne incognita* J2, after 24 and 48 h exposure at different filtrate concentrations, at 26 °C. The controls were a 50% dilution of the culture medium used for the fungal growth, without filtrate addition. Bars show the means ± SD. Asterisks show the means significantly different from the control at *p* ≤ 0.05 (*), *p* ≤ 0.01 (**) and *p* ≤ 0.001 (***), as indicated by Student’s *t* test.

Two in vitro assays carried out with different filtrate concentrations with isolates T7 and T26 showed an induction of the significantly higher second stage juveniles’ (J2) mortality, after 24 h exposure (Figure 2). This effect was evident for both isolates also at 48 h exposure at the lowest concentration tested (Figure 3). For the higher concentrations (10%, 25% and 50%), only the results for 24 h are shown as the data at 48 h were not significantly different.

**Figure 4:**
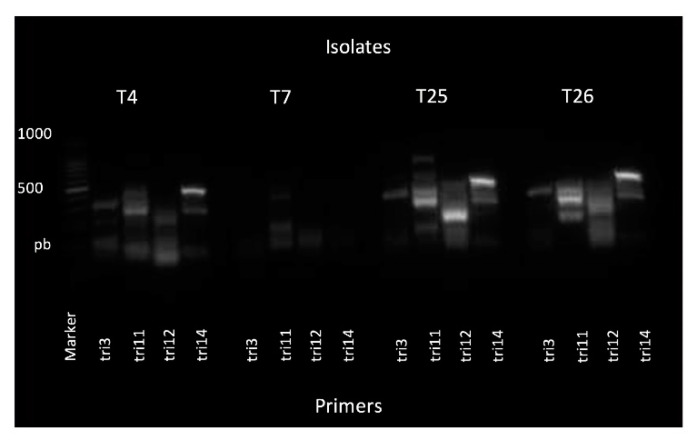
Gel electrophoresis of the PCR products obtained from *Trichoderma afroharzianum* (isolate T-7), *T. atroviride* (isolates T4 and T25) and *T. harzianum* (isolate T26), using the primer pairs corresponding to the trichothecene genes *Tri*-3, *Tri*-11, *Tri*-12 and *Tri*-14.

The PCR-amplified products of the four *TRI* genes were obtained from the isolates T4, T25 and T26, but not T7, which only showed a faint band for the *TRI-11* gene (Figure 4). The bands were eluted and sequenced. The BLAST analyses showed that they clustered with a *Trichoderma harzianum* hypothetical protein, with a high (>98%) nt-similarity (Table 2).

BLAST analyses of the 16S sequences obtained by PCR from the bacterial isolates obtained from the cysts showed variable identities with genera *Brucella, Rhizobium, Stenotrophomonas* and *Bacillus*, allowing their preliminary identification as members of these clades (Table 3).

## 3. Discussion

Hyphomycetes reported from PPNs include, among others, specialized egg parasites such as *P. chlamydosporia*, as well as saprotrophic or unspecialized *Fusarium* spp. [5,22,23,24]. However, the practical use of these fungi has been mostly limited to a number of isolates of *P. chlamydosporia* that consistently showed efficacy in PPN biocontrol, either in greenhouse or field conditions [5]. In spite of their prevalence and occurrence, the exploitation of *Fusarium* spp. isolates does not appear instead as a feasible practice, due to their proximity or even co-speciation with severe phytopathogenic members of this clade.

*Trichoderma* spp. have instead consistently shown a high potential for pest and disease management. Several species are known to act against soil-borne pathogens by different mechanisms including mycoparasitism, antibiosis and competition, whereas many isolates have been reported as PPN biocontrol agents or as plant-growth promoters [25,26,27,28,29]. *Trichoderma harzianum* has been applied to manage species such as *Meloidogyne* spp. [26,27] or *G. pallida* [30,31]. A direct in vitro activity of a *T. longibrachiatum* isolate on eggs and J2 of *H. avenae* was also reported [32]. The fungus penetrated the eggs, destroying their content, whereas the conidia germinated on the emerging J2, that were eventually parasitized. This effect was also observed in greenhouse assays, with significant effects on the nematodes density [32].

*Trichoderma afroharzianum* and *T. hirsutum* represent new reports from Algeria. Members of *Trichoderma* have been reported from Morocco as antagonists of *Sclerotium rolfsii* and other fungi [33,34,35], and from different agroclimatic areas in Tunisia [36]. In Algeria, *T. harzianum*, *T. atroviride* and *T. longibrachiatum* have been reported as antagonistic towards *Fusarium oxysporum* f. sp. *ciceris* [37], other *Fusarium* spp. associated to wheat head blight [38], and nematodes [19]. Other *Trichoderma* spp. have also been reported as antagonistic towards Fusarium wheat crown pathogens [39] and *F. oxysporum*, associated with tomato vascular diseases [40].

Due to their properties, many *Trichoderma* spp. isolates are the basic ingredients of commercial bioformulations applied to protect plants and/or enhance their growth [41,42,43,44]. Root colonization by *Trichoderma* spp. was found to induce metabolic changes such as the production of pathogenesis-related proteins in roots, thus increasing their resistance to pathogens [45,46].

Previous studies carried out with some *Trichoderma* spp. isolates from Algeria showed they are effective against PPN. In a first assay with filtrates, a *T. harzianum* isolate reduced the survival of *M. incognita* J2, with an effect comparable to that of a nematocidal treatment [19]. Many isolates produce primary or secondary metabolites and enzymes with diverse effects, important for industry and agriculture applications. Among them, mycotoxins such as trichothecenes have attracted much attention due to their detrimental effects on plant, animal or human health [47,48]. The production of trichotecenes and other active compounds by *Trichoderma* spp. isolates is related to their antimicrobial or biocontrol activities [49,50,51]. Data from this study confirm the nematotoxic activity of the *Trichoderma* filtrates tested, likely related to the release of trichotecenes, also supported by the identification of members of the *TRI* gene cluster in the isolates. Although not directly shown by the assay, it is likely that the J2 mortality induced by the filtrates in the in vitro tests was related to the production of one or more of such mycotoxins, known to exert a nematocidal effect [6,52,53].

As concerns the bacteria observed on PPNs, the BLAST analyses did not show close similarities of the PCR products obtained with known bacterial species, suggesting that the isolates require further investigations. However, the association of members close or belonging to genera *Brucella*, *Rhizobium* and *Stenotrophomonas* with cysts of *G*. *rostochiensis* was observed for the first time. Several *Bacillus* spp. have been reported in association to PPNs worldwide, and it is likely that, due to the wide diversity of this bacterial clade, new species or taxa specialized in nematode parasitism or plant growth promotion may be discovered in newly sampled areas [54,55,56].

Further testing and selection are needed for fungi and bacteria associated with *G. rostochiensis* and *Meloidogyne* sp. in Algeria, for the development of biological control bioformulations as an alternative to chemicals. It may be worth checking if the microorganisms suitable as biopesticides can also be effective against other phytopathogenic fungi and bacteria, for use where chemical treatments are applied improperly or result too expensive for farmers in Africa.

## 4. Materials and Methods

### 4.1. Isolation of Microbial Antagonists

The nematode-associated fungi were isolated from the soil, cysts of *G*. *rostochiensis* or females of *Meloidogyne* spp. collected during the period October–July during 2015, 2016 and 2017, from potato, tomato and olive plants. The surveys were carried out in some regions of western (Mostaganem), central (Algiers, Boumerdes), eastern (Bouira), northern and southern (El Oued) Algeria (Table 4).

The soil samples were collected with an auger from the plant rhizosphere at 10–30 cm depth. Each soil sample was a composite of several soil cores for a total of 1.5 kg. To extract the cysts nematodes, the soil samples were processed according to Fenwick’s method and then stored in a refrigerator at 4 °C until nematode extraction. Females of *Meloidogyne* spp. were extracted from roots under a stereoscope using a lanceolate needle. The cysts and females were transferred consecutively into a 0.1% NaOCl solution for 5 min, in streptomycin at a concentration of 100 μL/L for 15 min, and in sterile distilled water for 5 min for surface disinfection, and partially air-dried afterwards. Five surface-dried disinfected cysts or females were then placed onto the corners at a sterilized square cover glass which was placed on water agar in a Petri dish under sterile conditions. The plates were then incubated at 22 °C under continuous light, until the growth of fungal and bacterial colonies.

The antagonists were also directly isolated from the soil by sprinkling ca 1 g soil on the culture media and incubating the dishes until fungi and/or bacteria emerged. Alternatively, the method described by Elad [57] was used, by mixing ten grams of soil from each sample with 90 mL of sterile water through magnetic shaking for 1 h to obtain a good separation of particles. For variable propagule concentrations, series of dilutions were made from a stock solution from 10^−1^ to 10^−9^ in hemolysis tubes, then spreading the diluted aliquots on growing media incubated in the dark at 26 °C for 2 days (bacteria), or 7 days (fungi).

When the fungal colonies emerged, they were transferred to a Petri dish containing the agar medium (Potato Dextrose Agar: PDA, Corn Meal Agar: CMA or Czapeck) for growth, whereas the bacteria were transferred on nutrient agar media. The CMA and Czapeck culture media, conducive to the development of predatory fungi, were used for the isolation and production of fungal monosporic cultures.

The bacterial isolates were obtained from 0.1% NaOCl surface-sterilized disrupted cysts, adding a 10^−2^ suspension diluted in sterile distilled water on nutrient agar (NA). The plates were then stored at 22–24 °C in the dark, before collecting single cell colonies. All the fungal and bacterial isolates are stored at the Department of Botany of the Higher National School of Agronomy (ENSA), in El-Harrach, Algeria.

### 4.2. Molecular Identification

#### 4.2.1. DNA Extraction

The extraction of DNA was performed from mycelium using the Plant/Fungi DNA isolation kit (Norgen Biotek Corp., Canada), following the manufacturer’s instructions, or manually, using the cetyl-trimethyl ammonium bromide procedure (CTAB). The cells were obtained from pure in vitro cultures maintained in Petri dishes with PDA medium, and for the CTAB extraction only, they were fragmented in a vortex with a few, 400 µm diam. glass beads added (Sigma-Aldrich, St. Louis, MO, USA), for 5 min.

#### 4.2.2. ITS Sequencing

The internal transcribed spacer (ITS1-5.8S-ITS2) ribosomal gene region was amplified with the primers ITS1 and ITS4 [58] on a BioRad T100 Thermal cycler (BioRad, Hercules, CA, USA). The PCR reaction volume was 25 µL containing 2.5 μL 10X DreamTaq™ Green Buffer, 200 μM each deoxynucleotide triphosphates (dNTPs), 1 mM of both forward and reverse primers, 2 U/µL DreamTaq Hot Start DNA Polymerase (ThermoFisher Scientific, USA) and 2 µL (100 ng) of DNA of each fungus extract as template. PCR amplifications were carried out with the following protocol: 95 °C for 5 min (1 cycle), 95° C for 30 s, 55 °C for 30 s and 72 °C for 1 min (35 cycles), 72 °C for 7 min, and 12° C for storage. The DNA was purified and then sequenced by a commercial provider (Macrogen^TM^, Spain).

#### 4.2.3. RpbII Sequencing

To confirm the identification of isolates at the species level, the sequence of the RNA polymerase II subunit (*Rpb2*) gene was also used [59,60]. PCR reactions were set up using the same reagents and amounts described above, 1 μL of template (50 ng) and nuclease-free water, up to a total of 25 μL per reaction. The forward and reverse primers used were fRPB2-5F 5′-GA(T/C)GA(T/C)(A/C) G(A/T)GATCA(T/C)TT(T/C)GG-3′ and fRPB2-7cR 5′-CCCAT(A/G)GCTTG(T/C)TT (A/G)CCCAT-3′, respectively [59,61]. PCR was performed in a BioRad T100 Thermal cycler (BioRad, Hercules, CA, USA) with the following cycles: 1 initial cycle at 95 °C for 5 min; 30 cycles of 1 min at 95 °C, 2 min at 55 °C and 2 min at 72 °C, and a single final extension cycle at 72 °C for 10 min. The PCR products were examined on 1.8% agarose gels, purifying the fragments of the expected size with a commercial kit (GeneAll Expin^TM^ Combo GP kit) for extraction, then ligated to pGEM^®^-T Easy Vector Systems, for 16 h at 4 °C and cloned in *E. coli* JM109 Competent Cells (Promega, Madison, WI, USA), as recommended by the manufacturer. The recombinant clones with bands of the expected size were then sequenced by a commercial service (Macrogen, Madrid, Spain).

For the PCR amplification of bacterial 16S rRNA, the cells were collected with a spatula from 48-hr cultures growing on nutrient agar and kept at 26 °C. The DNA extraction was performed using a Bacterial Genomic DNA isolation kit (Norgen Biotek Corp., Canada). The PCR reaction volume was 25 µL, containing a final concentration of 10X PCR buffer, 200 μM each dNTPs, 1 mM of both 27F forward and 1492R reverse primers [62], 2 U/µL DreamTaq Hot Start DNA Polymerase (ThermoFisher Scientific, Waltham, MA, USA) and 2 µL of each bacterial culture extract as template. The 16S ribosomal gene was amplified with the following conditions: 95 °C for 5 min (1 cycle), 95 °C for 30 s (35 cycles), 49 °C for 30 s (35 cycles), and 72 °C for 1 min (35 cycles), 72 °C for 7 min, and 12 °C for storage. The quality of the PCR products was monitored on 1.5% Tris agarose gel, visualizing the bands by staining with Gel Red^TM^ N0ucleic Acid Gel Stain (Biotium, Fremont, CA, USA) with an Electrophoresis and Gel Documentation and Analysis System (Kodak, Rochester, NY, USA). The DNA was then eluted and sequenced by a commercial provider (Macrogen, Madrid, Spain).

### 4.3. In Vitro Assays

Second stage juveniles (J2) of the root-knot nematode *M. incognita* obtained from a greenhouse population maintained on cherry tomato were used to check the nematicidal effect of *Trichoderma* spp. filtrates. The nematodes were extracted from infested tomato root fragments maintained in tap water and aerated with a peristaltic pump in a flask. The J2 hatched from the eggs in the areated suspension were counted under a stereoscope and picked up using an eye lash, washed in sterile distilled water in a watch glass, and then individually added to the suspensions to be tested, in watch glasses. The watch glasses were kept in Petri dishes with a paper fragment saturated with water, to prevent drying, and stored at 26 °C for 24 or 48 h, in the dark.

The fungi used in the assay were: isolate T7, identified as *T. afroharzianum* and proceeding from *G. rostochiensis*-infested soil, and isolate T26, identified as *T*. *harzianum*, obtained from the cysts. The isolates were maintained on PDA and then incubated at 26 °C for 7 days, for filtrates preparation. Three mycelial plugs (6 mm diam.) were taken from each 7 day-old culture of each isolate and placed in an Erlenmeyer flask containing the Czapek Dox broth (Oxoid, Basingstoke, UK) liquid medium. After stirring for 2 days, the different filtrate concentrations (1%, 2.5%, 5%, 10%, 25% and 50%) were tested, placing approximately 20 J2 in each watch glass with the eye lash. Three replicates were performed for each treatment, with six concentrations, plus the control (50% of broth medium alone). After 24 and 48 h, the J2 mortality was checked by counting the living and dead *M. incognita*. Living J2 were scored after adding a droplet of 25% lactic acid to the watch glass, eventually counting under a stereoscope the number of nematodes that responded to the fast pH change, as revealed by their motility.

### 4.4. Trichothecene Gene Sequencing

To evaluate the presence of members of the *tri* gene cluster, coding for trichothecene biosynthesis in *Trichoderma* spp., the sequence of the gene *TRI* was used [63]. PCR reactions were set up using the same reagents and amounts described above, 1 μL of the template (50 ng) and nuclease-free water, up to a total of 25 μL per reaction. Four pairs of forward and reverse primers: (*TRI3*, *TRI11*, *TRI12* AND *TRI14*) were tested, on the *Trichoderma* isolates T4, T7, T25 and T26 (Table 5). PCR was performed in a BioRad T100 Thermal cycler (BioRad, Hercules, CA, USA) following the indications described above. The quality of the PCR products was monitored on 1.5% Tris agarose gel, visualizing the bands by staining with Gel Red^TM^ Nucleic Acid Gel Stain (Biotium, Fremont, CA, USA) with an Electrophoresis and Gel Documentation and Analysis System (Kodak, Rochester, NY, USA). The DNA was then eluted and sequenced by a commercial provider (Macrogen, Madrid, Spain).

### 4.5. Phylogenetic Analyses

The ITS1-2 and rpb2 sequences produced were validated by comparison with those available in GenBank using the BLAST analysis online tool. Multiple sequence alignments were constructed with a Windows^TM^ interface for CLUSTAL W [64] provided by BioEdit ver. 7.0.1, with default parameters [65], using the sequences produced with the closest entries retrieved from GenBank for *Trichoderma* spp. The evolutionary distance of the taxa was inferred with MEGA X [66] using the trimmed alignments, eliminating the positions with external gaps or missing data. Phylogenetic trees were constructed with the neighbor-joining method [67] and the Jukes–Cantor metrics, applying the pairwise deletion of ambiguous positions (ITS1-2 region) or deleting all the positions with gaps and missing data (*Rpb2* gene) [68,69].

## Figures and Tables

**Table 1:** List of the fungi isolated from the cysts of *Globodera rostochiensis*, the females of *Meloidogyne* spp. or rhizosphere soil, from food crops sampled in Algeria.

Isolate Code ^a^	Source ^a^	Location and Crop ^a^	Identification ^b^/Phenotype ^c^
T1, T2, T3, T11, T18, T19	C	El Oued, potato	*Trichoderma harzianum* ^b^
T4	C	Boumerdes, potato	*T. harzianum* ^b^
T8, T24	C	Bouira, potato	*T. harzianum* ^b^
T9, T28	S	Bouira, olive	*T. harzianum* ^b^
T12, T20, T21, T23, T27	F	Tipaza, tomato	*T. harzianum* ^b^
T15	C	El Oued, potato	*T. harzianum*
T17	C	Bouira, potato	*T. harzianum* ^b^
T26	C	El Oued, potato	*T. harzianum* ^b^
T14	C	Bouira, potato	*T. harzianum* group—*T. afroharzianum* ^b,§^
T5	C	El Oued, potato	*T. afroharzianum* ^b^
T6, T16	C	Mostaganem, potato	*T. afroharzianum* ^b^
T7	S	Tipaza, potato	*T. afroharzianum* ^b^
T10	S	Tipaza, tomato	*T. afroharzianum* ^b^
T22	C	Bouira, potato	*T. afroharzianum* ^b^
T25	S	ENSA, tomato	*T. atroviride* ^b^
T29	S	Tipaza, tomato	*T. hirsutum* ^b^
K10-1	C	Bouira, potato	*Fusarium* sp./short conidiophore
K10-2	C	Bouira, potato	*Fusarium* sp./microconidia, pycnidia
K10-3	C	Bouira, potato	*Fusarium oxysporum*
K10-4	C	Bouira, potato	*Fusarium* sp./macroconidia
KB1, KB4(2), KB6′, KB7	C	Boumerdes, potato	*Fusarium* spp.
KB8	C	Boumerdes, potato	*Fusarium* sp./macro and microconidia
KB2	C	Boumerdes, potato	*F. culmorum*
KB5	C	Boumerdes, potato	*Fusarium* sp./microconidia
KOK(4)	C	El Oued, potato	*F. solani*
KO3	C	El Oued, potato	*F. avenaceum*/macro and microconidia
F-ox	C	El Oued, potato	*F. oxysporum*
SKM	S	ENSA (Algiers)	*F. oxysporum*
f-cz	S	Tipaza, potato	*Fusarium* sp.
F42-3	F	Tipaza, tomato	*Fusarium* sp./microconidia
F-CMA	S	Tipaza, potato	*Arthrobotrys* sp.
F42-4, FT5	F	Tipaza, tomato	*Alternaria* sp.
T13	S	Mostaganem, potato	*Penicillium* sp.

^a^ Source: s = soil; c = cyst of *G. rostochiensis*; f = *Meloidogyne* spp. adult female. ^b^ Identification based on internal transcribed spacer (ITS)1-2 and/or *Rpb2* gene sequence data (see Figure 1). ^c^ Phenotypic traits are shown after “/”. ^§^ Unresolved identification.

**Table 2:** BLAST best matches of the sequenced *Tri* genes amplicons from the *Trichoderma* spp. isolates.

Isolate	Putative Target Gene	Length (nt)	BLAST Accessions	Identity (%)	Query Cover (%)
T4	*TRI3*	269	XM_024915040.1	98.02	73.0
T4	*TRI3*	645	XM_024923427.1	99.69	98.0
T4	*TRI14*	436	XM_024921455.1	99.06	97.0
T25	*TRI3*	645	XM_024923427.1	99.69	98.0
T25	*TRI11*	1218	XM_024912120.1	96.59	52.0
T25	*TRI14*	436	XM_024921455.1	99.06	97.0
T26	*TRI3*	269	XM_024915040.1	98.02	73.0
T26	*TRI14*	436	XM_024921455.1	99.06	97.0

**Table 3:** Bacterial isolates found in the cysts of *Globodera rostochiensis* from Algeria, and the closest results of the BLAST NCBI queries for the PCR-amplified 16S rRNA ribosomal gene sequences.

Isolates	Original Locations	Closest GenBank acceSsions and Genera	BLAST Query Cover (%)	BLAST Identity (%)
DigB	Bouira	KJ634686.1 *Brucella*	76	91.87
NaKB	Bouira	EU748920.1 *Rhizobium*	29	78.67
NaB	Bouira	KY079269.1 *Stenotrophomonas*	23	86.49
Kj	Mostaganem	MH497609.1 *Bacillus*	40	87.84

**Table 4:** Geo-ecological description with the location of sampling sites.

Localities	Bioclimatic Classificaton	Annual Rainfall (mm)	Mean Annual Air Temperature (°C)	Longitude (E)	Latitude (N)
Alger Boumerdes Tipaza	Humid Humid to Sub-humid Sub-humid	707 739 631	17.7 18 18.5	3°02′31″ 3°28′38″ 2°26′26.76″	36°45′08″ 36°45′00″ 36°35′42.23″
East: Bouira	Semi-arid	659	16.5	3°54′07″	36°22′29″
West: Mostaganem	Semi arid	347	17.9	0°33′21″	35°44′14″
South: ElOud	Desert	74	25	6°52′03”	33°22′06″

**Table 5:** Target trichotecene *TRI* genes, primers and sequences used for the PCR amplification.

Gene	Primer	Sequence (5′-3′)
*TRI14*	T14int5 T14int	CACAGGTGTTACTGAGCT CCAGCATAAGTGCCATTG
*TRI12*	T12int5 T12int3	CAACGTTATAGCGACAGG AACGCAGCAGTGAAGATC
*TRI11*	T11int3 T11int5b	CCCACAAGAAGTGTGTCT CGTTGCAGTACAACTCGT
*TRI3*	T3int3 T3int5	CCTCCTCCTGACTGTAAT TATTGAGGAGCTGCGAGA
